# Sexology, Popular Science and Queer History in *Anders als die Andern (Different from the Others)*

**DOI:** 10.1111/1468-0424.12381

**Published:** 2018-09-24

**Authors:** Ina Linge

In 1919, the same as year *Anders als die Andern* (*Different from the Others*) was released in cinemas all over Germany, the Berlin sexologist Magnus Hirschfeld exclaimed that ‘those who work to educate people about sexual matters not only have the right, but the duty to use film, in addition to the spoken and written word’.^1^ Film apparently offered an alternative way to transmit ideas, but what could the new medium of film achieve that other forms of dissemination, such as lectures, public talks, monographs, scientific journals or popular science books could not? In this article, I investigate how sexology looks different once we understand it in the context of the institution of cinema and the medium of film. In doing so, I examine the contributions, but also the challenges, risks and difficulties of this new medium for the sexological project.^2^

*Different from the Others* was directed by the Austrian film‐maker Richard Oswald, in collaboration with Magnus Hirschfeld. The film is said to be the first of its kind to explicitly and sympathetically portray homosexuality and to demand the decriminalisation of homosexual acts between men.^3^ *Different from the Others* follows the tragic events in the life of Paul Körner (Conrad Veidt), his relationship with his student, Kurt Sivers (Fritz Schulz), his encounter with the blackmailer Franz Bollek (Reinhold Schünzel), Körner's public humiliation and finally his death by suicide. The climactic moment of the film is a lecture given by an unnamed sexologist (and portrayed by Magnus Hirschfeld himself), which explains the plight of sexual minorities. The lecture is attended by Körner, who is accompanied by Kurt's sister, Else (Anita Berber).

Today, the film exists only in fragments.^4^ Of the original, approximately ninety minutes in length, only fifty‐one minutes remain. The reconstructed film is based primarily on a fragment of the re‐edited, abridged version of *Different from the Others* entitled *Gesetze der Liebe: Aus der Mappe eines Sexualforscher*s (*Laws of Love: From the Portfolio of a Sexologist*), a 1927 anthology work consisting of several short films, which re‐used parts from *Different from the Others* and which was preserved by Gosfilmofond in the former USSR.^5^ This film is important for our understanding of the relationship between film and sexology because, despite its fragmented nature, it is the only remaining example of a collaborative film project between a sexologist and a film‐maker. While the following article will examine the opportunities and challenges offered by the medium of film in this particular and famous example, I hope to begin a conversation about the contribution of film to the sexological project at large.

The article falls into two parts. Implied in sexology's turn to film is its desire for popularisation: cinema and the new genre of the *Aufklärungsfilm* (sexual enlightenment film) as educational mass medium show the popular side of sexology. The first part of this article will investigate what opportunities and possibilities Hirschfeld saw in film for this particular purpose. By comparing *Different from the Others* with Hirschfeld's previous work in popular science, two common aims are shown: to reach a broad audience, and to ‘usualise’ cultural spheres of the ‘third sex’, a term commonly used in sexological work and subculture to describe same‐sex desiring as well as gender‐variant people. However, using film to communicate these aims altered each in unexpected and contradictory ways. The second part of the article will focus on what happens when a common strategy employed by sexologists – to use history affirmatively to secure identity categories – is represented in the visual medium of film. Here, building on the argument of the first section, the article investigates how film enhances, complicates and contradicts a core aim of Hirschfeld's particular brand of sexology. In this way, I intend to show that the complexity of film served to hold up a mirror to sexology's hidden complexities, threats and losses.

## Popular science: *Berlin's Third Sex* and *Different from the Others*

Current scholarship about sexology is experiencing a turning point. Until very recently, the dominant assumption among historians and scholars of neighbouring disciplines was that sexology existed as a medical field of knowledge that was clearly understood as such by its contemporaries.^6^ In recent years, however, some scholars have begun to explore the cross‐disciplinary nature of sexology and how other forms of knowledge and other modes of scholarly enquiry, such as literature, life writing, history and ethnography (to name just a few), significantly shaped the sexual sciences, beyond the domain of the medical.^7^ Sexology also went beyond that field in the sense that sexologists tried to reach new audiences, beyond medical professionals. This included dialogue with patients, efforts to educate the public about sexual matters, and the discussion of sexological matters in magazines and popular journals. As such, with this turn in sexological scholarship in mind, sexology should be understood as a cross‐disciplinary endeavour which was not clearly defined and which drew on various alliances across disciplinary and methodological boundaries, thus engaging a variety of audiences.

Magnus Hirschfeld (1868–1935) was one of the most influential sexologists, and one whose work took place at the intersection between science and sexual rights. As activist, writer, publisher, and practitioner and founder of the first Institute of Sexology, Hirschfeld was a prominent public figure in early twentieth‐century Germany and beyond, notoriously dubbed the ‘Einstein of Sex’ by the US media during his lecture visit in 1930.^8^ His most well‐known scientific contribution to the study of sexology was his *Zwischenstufenlehre*, the theory or study of sexual intermediacy, which argued that any form of sexual intermediacy was part of the natural constitution of body and mind, rather than acquired through seduction or the result of degeneracy.^9^ Sexual intermediates not only included homosexual men and women, but all forms of gender and sexual variance, including ‘transvestitism’ – a term which he coined – and ‘hermaphroditism’.^10^ Hirschfeld's lifelong commitment to the legal rights and protection of homosexual men was formalised by the co‐founding of the ‘Wissenschaftlich‐humanitäres Komitee’ (WhK), the scientific humanitarian committee, in Berlin in May 1897.^11^ As Germany's first gay rights lobby, the WhK's priority was to petition the government to decriminalise homosexuality by abolishing §175 of the German Penal Code, which made (male) homosexuality a crime.

For Hirschfeld, film provided another format – in addition to pamphlets and lectures – to promote his campaign against §175. With the end of the First World War, the abolition of monarchy, the proclamation of the new Republic on 9 November 1918, the following months of political upheaval, and also the promise of a reformed Penal Code, Hirschfeld and his allies saw a chance to once again rally against the paragraph.^12^ The pre‐war public scandals around homosexuality, especially the Eulenburg scandal from 1906, when the journalist Maximilian Harden accused Prince Philipp Eulenburg, a close confidant of Emperor Wilhelm II, of homosexuality, though detrimental for Hirschfeld's efforts to repeal §175, had also taught him the power of public opinion.^13^ The extensive sociocultural apparatus of cinema offered an excellent way to reach large audiences: in 1919, the year *Different from the Others* was released, over 200 production companies released a combined 500 films in Germany. There were 3,000 cinemas across the country. One million people went to the cinema every day.^14^ The release of *Different from the Others* in 1919 was the start of an auspicious year for Hirschfeld: the inauguration of his Institute of Sexology took place on 6 July 1919 and the modern Weimar Constitution, which no longer enforced media censorship, came into force on 11 August 1919.

This intention to publicise science to educate the public is tightly linked to the circumstances of modernity. The increasing reliance on science in all aspects of life yielded hopes about a modernised future. But the difference in knowledge between scientific experts and lay people also triggered scepticism and mistrust in a modernised world that relied on scientific knowledge without fully understanding it. Popular science attempts to establish communication between experts and the rest of society.^15^

Publicising sexological knowledge is not unique to Hirschfeld's engagement with film, but is in line with his previous work to bridge the gap between experts and laypeople. In his 1904 publication *Berlins drittes Geschlecht* (*Berlin's Third Sex*), Hirschfeld takes his readers on a journey through Berlin's third sex subcultural scene. The book appeared in a series called *Großstadt‐Dokumente* (metropolis documents) edited by Hans Ostwald. The series included sociological studies of fringe and marginalised groups that were otherwise not available to study and was praised by social scientists, as well as enjoying general popularity. Much like *Different from the Others*, *Berlin's Third Sex* is clearly addressed to a general audience. At seventy‐seven pages, the book is much shorter than many of Hirschfeld's scientific tomes and clearly designed for broad dissemination. In the foreword, Hirschfeld explains his reasons for writing a book for a general audience:

While the results of my research into the field of homosexuality have only been published in specialist journals to date … it has long been clear to me that knowledge of an area that is intertwined with the interests of so many families, of every class, would not and could not remain forever confined in the closed community of specialists or academic circles.^16^

Here, Hirschfeld's argument reflects the fundamental premise of his sexological work. If sexual diversity is a fundamental part of all human life, regardless of dividing categories such as class, then everyone should have access to adequate information and education about sexual matters, free from stigma and prejudice. This is what Hirschfeld also claims for film in his speech cited at the beginning of this article.

In addition to a shared desire for a broad, non‐specialist audience, *Berlin's Third Sex* and *Different from the Others* share an intention to ‘usualise’ third sex experience. In *Berlin's Third Sex*, Hirschfeld takes his readers on an exploration of Berlin's underworld of ‘regular gatherings held by homosexuals on certain evenings in certain bars’, as well as other localities, including ‘restaurants, hotels, pensions, bathing facilities and pleasure spots’.^17^ Examples of precisely such meeting places can be seen in *Different from the Others*, too. Here, the audience witnesses two scenes of third sex spaces. The first is a ball, attended both by Körner and his blackmailer. The second is a club or café, where Körner's blackmailer awaits correspondence from his victim. In both *Berlin's Third Sex* and *Different from the Others*, these glimpses serve to ‘normalise’ third sex experience, or, to use a less value‐laden expression that more adequately represents Hirschfeld's agenda, they serve to ‘usualise’ sexual intermediacy by showing the everydayness and frequency with which it occurs, something Hirschfeld was very keen to show in his other work, too. Throughout his career, he was keen to collect statistics about the prevalence of homosexuality. Thus, in 1904, he published an essay in his *Jahrbuch für sexuelle Zwischenstufen* (*Yearbook for Sexual Intermediacy*), which argued that homosexuals made up more than 2 per cent of the German population.^18^ Hirschfeld also authored a psycho‐biological questionnaire which his patients at the Institute of Sexology were asked to complete. Although exact numbers cannot be stated for certain, Hirschfeld is said to have collected over 40,000 completed questionnaires.^19^ Both *Berlin's Third Sex* and *Different from the Others* add to this project in important ways. Not only do they attempt to draw the attention of the intended heterosexual audience and readership to the fact that sexual intermediaries exist in their midst, but that intermediates live ‘normal’ lives with friendship circles and cultural events that are similar to those of the heterosexual audience and readership.

Highlighting the above‐mentioned similarities between *Berlin's Third Sex* and *Different from the Others* – to reach a broad audience, and to ‘usualise’ third sex culture – what appears important about the visual dimension in *Different from the Others* is not so much the translation of sexological knowledge into the visual medium per se, but rather the much broader audience that can be addressed via film. Visibility is significant only in a figurative sense, as political visibility, and hence as a counter‐force to societal ignorance, in that it acknowledges that individuals like Körner do exist, do fall prey to the harmful §175, and that their fate should not be ignored by the society in which they live.

Despite the obvious shared goals of *Berlin's Third Sex* and *Different from the Others*, however, the turn towards film had unforeseen consequences. To begin with, the timing of Hirschfeld's interest in film created a particular context for the topic of sexual intermediacy that distracted from the film's goal to arouse pity for the plight of homosexuals. Hirschfeld's turn towards educational film coincided with a wave of *Aufklärungsfilme*, enlightenment films or sex education films. The *Aufklärungsfilm* was an already familiar genre present in pre‐war films in Europe, as evidenced, for example, in the Danish film *Den hvide Slavehandel *(*The White Slave Trade*) from 1910. Proponents of the social hygiene movement soon realised that film could be enlisted to reach the public in new ways, beyond the use of pamphlets and lectures. This film genre was then picked up in Germany during the interwar years. It was in this climate that the film‐maker Richard Oswald rose to fame. Oswald became a prolific film‐maker, working in a variety of genres, producing adaptations of crime and romance literature, social melodramas and epic historical dramas.^20^ Yet, it was the genre of the *Aufklärungsfilm*, which Oswald himself called ‘social hygiene film’ (sozialhygienisches Filmwerk), that would make him famous. Under Oswald's direction, hygiene films took on a unique shape. As Jill Smith summarises, Oswald's experience with entertainment cinema taught him that ‘hygiene films first had to *appeal* to the viewing public before they could *educate* them’.^21^ Consequently, sexual hygiene films ‘became the prototype for a new symbiosis of science and film, of public health advocacy and entertainment’.^22^

It was in the context of the *Aufklärungsfilm* that Oswald began to collaborate with sexologists and professional bodies. In 1917 Oswald released *Es werde Licht!* (*Let There Be Light!*, lost) about the dangers of syphilis. During the production of *Es werde Licht!*, the German Society for Combating Venereal Diseases acted as scientific consultant. Oswald produced several sequels for the film. The technical advisor for Parts 2 and 3 (both lost) was Iwan Bloch, who, together with Hirschfeld, is considered one of the founders of sexology.^23^ Under his supervision, these two sequels were released with the endorsement of the Medical Society for Sexology.^24^ In 1918, Hirschfeld, too, became interested in the potential of film. He took over the role of scientific advisor for the fourth and final sequel, entitled *Sündige Mütter* (*Guilty Mothers*, lost), which explores the consequences of §218 of the German Criminal Code outlawing abortion. In 1919 Oswald and Hirschfeld collaborated on two further films, *Die Prostitution* (lost) and *Different from the Others*.

The topic of homosexuality in *Different from the Others*, however, is at odds with other titles that form part of Oswald's social hygiene oeuvre. Topics such as syphilis, in which people became acutely aware of the prevalence of syphilis among soldiers and its dire consequences for the health of the nation especially in 1917 during the context of the ongoing war, as well as topics such as motherhood and sex work, were seen as issues in need of a broad (though appropriately mature) audience. One conservative reviewer of *Different from the Others*, the pastor Martin Cornils, comments that, in principle, he is not an opponent of the *Aufklärungsfilm*. He lauds Oswald's *Let There Be Light!* for its presentation of the dangers of venereal disease. He sees its presentation in film format justified, because such issues of normal sexual life concern all human beings. His critique of *Different from the Others* lies in the fact that the film depicts homosexuality, ‘a disease and anomaly concerning a small section of mankind. Its treatment cannot be decided on by a forum of cinema‐goers’.^25^ According to Cornils, the topic of sexual intermediacy did not seem to concern the majority of the heterosexual viewing public and was therefore deemed inappropriate. This was contrary to Hirschfeld's aims.

Moreover, opinions on the naturalness of homosexuality were far from unified. Hirschfeld's argument that homosexuality could not be criminalised because it occurred naturally and was therefore part of normal variation were radical and considered controversial by many. His theories contradicted various earlier and competing theories of homosexuality as pathological perversion (Richard von Krafft‐Ebing), acquired trait (Albert von Schrenk‐Notzing), hereditary disposition (Karl Friedrich Otto Westphal), or a form of evolutionary atavism, a return to a lower evolutionary and hermaphroditic stage (James G. Kiernan).^26^ As such, unlike other films in Oswald's social hygiene genre, *Different from the Others* depicted a more controversial political issue that divided expert opinion, and it did so for a potential audience of one million people to see, every day.

Add to this the fact that the *Aufklärungsfilm* was already a controversial form of cinema. Smith argues that Oswald's *Aufklärungsfilme* ‘must not be seen as products of the alleged commercialization of sexuality for belittled prurient masses but rather as genuine and influential contributions to the sexual‐policy discourses in the nascent democratic culture of the Weimar Republic’.^27^ Critics, however, did not necessarily agree with this evaluation. The self‐styled *Kinoreformer*, opponents of what they considered low‐brow and trashy films, were immensely critical of the new role of cinema as entertainer of the masses, fearing that cinema would negatively affect the morals and ethics of the public, as well as offer a watered‐down version of aesthetically established forms of art, such as literature. For some, it was not the institution of cinema itself that was dangerous, but so‐called *Schundfilme*, trash‐ or B‐movies, which served the sole purpose of titillating the audience, often with the help of sexually explicit content. For the *Kinoreformer*, cinema had the potential to educate the public and thereby serve a pedagogical purpose. From this point of view, *Aufklärungsfilme*, which provided a ‘unique blend of titillation and education’, were considered a perversion of the very idea of cinema.^28^

*Different from the Others* was ultimately censored and its audience severely restricted. In Germany, censorship had been officially abolished in 1918. While in theory this guaranteed full freedom of expression to the arts and sciences, in practice accusations of blasphemy and obscenity could still lead to criminal prosecution.^29^ When *Anders* premiered in Berlin on 24 May 1919, the film turned into a box office hit, but it caused uproar and scandal. Various attempts to have the film banned in the northern regions of Germany failed, but in the southern regions censorship was more readily used, based on laws prohibiting obscenity. On 16 June 1920, a new ‘Lichtspielgesetz’ went into law, which required a film to pass censorship before release, and on 16 October 1920, *Anders* was banned to the public and limited to screenings for specialist audiences. This ultimately limited the reach of the film and thwarted its primary aim to reach a broad audience. The 1927 version, *Laws of Love*, was also almost immediately banned, large portions removed and put under age‐restriction for adults only.^30^

**Figure 1:**
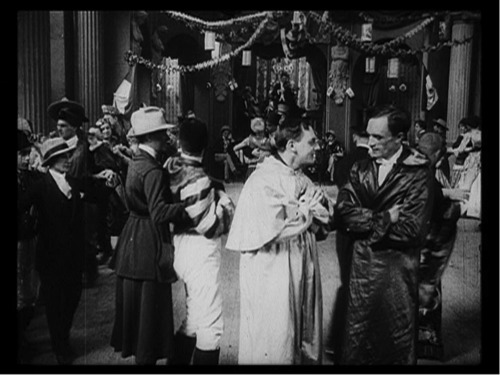
At the ball. Richard Oswald, *Anders als die Andern*, 1919. © Filmmuseum München.

This shift in context, which came about when Hirschfeld's sexual rights activism was presented in the form of film, also profoundly influenced other sexological aims. In particular, attempts to ‘usualise’ third sex culture were significantly altered when put in the context of the *Aufklärungsfilm*. Consider the scene in which Körner and Franz Bollek (who will later blackmail him) strike up a casual relationship at a homosexual ball (Figure 1). This scene reveals a high investment in the film's *mise en scène*, but one that is potentially at odds with the film's primary aim of raising awareness and pity. Men appear in dresses, dancing with other men in historical costume, two women are dressed in shirts, suits and braces, and various figures appear deliberately androgynous or cannot be read as clearly male or female. The lush costumes worn by the dancers make elaborate pageantry a key feature of queer experience. The round dance, at which Körner greedily fills a glass of champagne before taking Bollek home, evokes a sense of exuberance, flamboyance and alcohol‐fuelled debauchery. Even if these images are not, in themselves, necessarily explicit, the context spoke for itself. As Richard Dyer argues, ‘such images may have carried more of a frisson because people expected to see something thrilling’.^31^

The exuberant depiction of subcultural life could be understood as an attempt to put into visual form something that Hirschfeld also explored in *Berlin's Third Sex*. Here, he speaks of the ‘dual personality’ of many third sex Berliners:

The ease with which one can sink unseen into a city of two and a half million inhabitants greatly facilitates the dual personality so often found in the sexual arena. The professional self and the sexual self, day self and night self are often two utterly distinct personalities in one body, the one proud and honourable, most noble and conscientious, the other its antithesis. This applies to homosexuals as well as the normally sexed.^32^

This argumentative move to ‘usualise’ the homosexual is also executed in *Different from the Others*. The film features a scene in which the young Körner is taken to a brothel by his fellow students. Here, he is soon flanked by two women who pull him towards them and kiss him. Körner fends off their advances and escapes their embraces. The ‘normally sexed’, much like Körner, have a daytime and night‐time personality. In the context of the visual medium of film, however, the parallel between the brothel scene and the ball scene does not immediately call to mind Hirschfeld's description of dual personality, but draws the viewers’ attention to the parallel between homosexual and heterosexual carnal lust and promiscuity and implies the same feelings among the men and women at the ball scene. The visual parallel between the scenes also draws attention to the eroticism of the ball scene, which shows people in physical contact with one another and dancing cheek to cheek. This scene may serve to ‘usualise’ third sex experience, but it also casts an unintended light on lust and desire. *Berlin's Third Sex* also focuses on the underbelly of Berlin, but in the context of the *Großstadt‐Dokumente*, which provided sociological excursions into subcultures in order to make them available for further study, while the glimpse of third sex spaces in *Anders als die Andern* only strengthens accusations that *Aufklärungsfilme* are more titillating than educational.

If we view *Different from the Others* in the context of Hirschfeld's previous work, which sought to educate the public about matters pertaining to human sexuality and to show that scientific education opens the way to a fairer and more just society, Hirschfeld's turn towards film can be understood not so much as a turn towards the visual, but a further step into the popular. Analogous to his popular science book *Berlin's Third Sex*, *Different from the Others* aims to address a broad and non‐specialist audience and ‘usualise’ sexual intermediacy in the eyes of the audience by introducing it to the urban subcultural world of the third sex. Yet, when this popular science agenda is translated into the visual medium of film, it takes on a life of its own. The audience is meant to experience *Different from the Others* as an *Aufklärungsfilm*. In this context, it appeared more scientifically controversial and politically risky than previous films of this genre, and the foremost aim of wide dissemination was ultimately thwarted by censorship. Visualising the subcultural encounters of its protagonist and the dual personality of the city‐dweller drew unintended attention to lust and desire, rather than generating sympathy for homosexuals.

This discussion of the challenges of translating a popular science programme from the written text to the screen is not to say that *Different from the Others* is a failed film project, or that Hirschfeld misunderstood the medium of film. Rather, it highlights the complexity of this new medium, which may have been chosen by Hirschfeld to popularise his scientific views, but which altered intended aims in unexpected and contradictory ways.

## ‘An endless procession of them, from all times’: Representing queer history in film

**Figure 2:**
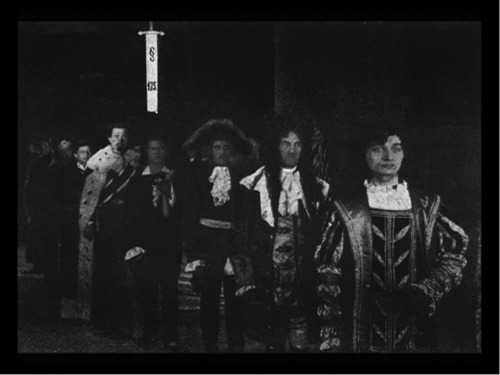
‘An endless procession …’. Richard Oswald, *Anders als die Andern*, 1919. © Filmmuseum München.

In *Different from the Others*, we do not exclusively encounter representatives of the third sex in the metropolitan underworld of queer costume balls and bars. The film also employs a more common strategy of representing the third sex as noble and virtuous. The film begins with a scene in which Paul Körner is reading newspaper reports about various cases of suicide amongst promising young men. As he comes to a realisation, Körner drops the newspaper. The vision that follows explains this: ‘Paul Körner senses a common thread’, the next intertitle reads. ‘The sword of Damocles that is §175 made life impossible for these unfortunate individuals. In his mind's eye he sees an endless procession of them, from all times and countries, passing in review’.^33^ In 1919, the audience would have then seen a long shot of a slow procession of men in period costumes. After Körner's death, he joins the procession as its final member. In the 2011 version of the film, a production still (Figure 2) replaces this historical vignette, which is now considered lost. The next intertitle further describes the procession: ‘The figures appearing in this procession include such luminaries as Peter Tchaikovsky, Leonardo da Vinci, Oscar Wilde, King Frederick II of Prussia, and King Ludwig II of Bavaria’.

Sexology was profoundly invested in the re‐telling of history. Some of the historical figures mentioned in *Different from the Others* had already been featured in Hirschfeld's earlier writing. He praised Freud's essay on Leonardo da Vinci, despite its critical take on his own sexological theories.^34^ In his *Yearbook*, Hirschfeld published biographical vignettes of famous homosexual poets and writers, including Oscar Wilde.^35^ These attempts at embedding sexual intermediacy in cultural history served to assure his contemporaries that they were part of a long and respectable lineage of sexual intermediates. Women, too, were not entirely excluded from this lineage: one of Hirschfeld's very first publications paid tribute to Sappho.^36^ This strategy to use an encounter with the historical past in order to produce queerness in the present abounds in the works of other sexologists, too, including Iwan Bloch and Albert Moll in Germany, and Edward Carpenter, Havelock Ellis and John Addington Symonds in Great Britain.^37^ Long before *Different from the Others* was released, famous regents from the German past had been used as standard examples to illustrate that homosexuality had existed across the ages. In *Die Entdeckung der Seele (The Discovery of the Soul)* from 1880, the zoologist‐cum‐hygienist, Gustav Jäger, lists Frederick the Great and King Ludwig II as examples of historical homosexuals. Similarly, the German psychiatrist and influential sexologist Albert Moll, who advocated for apolitical and impartial science, mentioned Ludwig II in a chapter on historical homosexuals.^38^ Robert D. Tobin argues that ‘the heavy reliance of nineteenth‐century thinkers on long lists of artists, writers, and philosophers as evidence for the existence and legitimacy of same‐sex desire came to stand in for a homosexual culture’, so much so that, ‘by the end of the century, these lists were commonplace’.^39^

Reference to historical homosexuality served one very specific purpose: to use the historical existence of sexual intermediates to justify the existence of sexual intermediacy in the present and to give hope for a future of tolerance and acceptance. In *Disturbing Practices*, Laura Doan describes attempts like these to uncover specific identity histories as an ‘ancestral genealogy’, or the gathering of ‘recovery histories’.^40^ Such identity histories, however, are necessarily constructed, shedding more light on the desire of the person recovering these histories than history itself. Any production of identity categories across time is necessarily teleological. After all, as Doan goes on to argue, not everyone in the early twentieth century understood themselves in terms of identity categories such as homosexual or heterosexual. The challenge, as Doan sees it, is to account for forms of experience in the past that cannot be clearly mapped onto contemporary identity categories.

On the face of it, *Different from the Others* seems fully committed to an effective history of ancestral genealogy. The historical scene in *Different from the Others* paints a picture of the homosexual as extraordinary and productive in order to visualise a proud ancestry that counteracts gay shame. The film claims that a series of well‐known historical figures are part of Körner's homosexual ancestry. The identities of the particular men visualised as belonging to a lineage of homosexual kinship include a playwright, a composer, a polymath, and kings famous for their contribution to music, architecture and nation‐building. This line of historical men serves as a decorous kind of Pride Parade, which emphasises the creativity and productivity of noble homosexual men in order to directly counter the shame associated with homosexuality. Homosexuality, here, becomes the distinguishing trait of extraordinary men, a badge of honour and respectability.^41^

This historical parade also serves to depict the lineage of homosexuals not as an anonymous mass, but as a number of individuals, to show the extent of harm done by homophobia, and to ask for compassion and pity. Neatly lined up in a procession, homosexuality is here presented as a stable identity in a lineage that can be directly traced through the ages and across cultures. But the formation of homosexual kinship as procession serves its own purpose. The homosexual procession simultaneously resembles a coronation, a military parade, a religious service or a funeral procession. All, of course, support the film's plot and its sexological message in some way. The coronation emphasises that some of the most powerful men in German history were (according to the list) homosexuals; military connotations reinforce the message that homosexual men could be just as virile as heterosexual men and contribute to the founding of a nation, as argued repeatedly by Symonds and Carpenter, for instance; sombre religiosity appeals to a predominantly Christian audience; and the sense of a funeral procession foreshadows Körner's fate and the life‐threatening danger of §175 (as well as connecting back to the series of suicide victims in the newspaper reports). Whereas depictions of religious or military events are not unusual in films from the 1910s, this procession in single file stands out. Modernist literature and film were fascinated but also deeply troubled by the masses. F. W. Murnau's *Faust* (1926) shows devout masses in a church, all facing the same direction (away from Faust, whose pact with Mephisto bars him from entering). Fritz Lang's *Metropolis* (1927) shows masses of workers turned towards Maria (Brigitte Helm). The emphasis here is on the uniformity of the masses as a coherent group that can be powerful but also destructive. The single file in *Different from the Others*, however, emphasises each man as he passes below the sword of Damocles, their faces in full view, foregrounding their individuality. This is decidedly not homosexuality as a mass, but as a sequence of individuals. The parade's resemblance to religious service and funeral procession serves to make an appeal to Christian values of compassion. When Körner joins the historical procession as its final member, the film demonstrates the long history of persecution and violence, that this violence persists in the present through §175, and that the post‐First‐World‐War world is not free from discrimination.

Finally, the historical vignette also serves to establish a potential for communal uprising as a vision for the future. Körner creates and recalls an imaginary kinship in order to make sense of his own situation. It is in Körner's imagination – ‘in his mind's eye’ – that past and present become linked. It is Körner's own understanding, his imagining of that past, which informs the present. This is not just an act of remembering history, but an active vision that appears to Körner and connects his own situation to circumstances of the past. It is just before his suicide that he imagines that ‘he himself now joins the file as its final member’. As much as the past invades the present, the present, here, infiltrates the past. As Körner joins the parade as its final member and Hirschfeld himself calls to action against §175 in the final scene of *Different from the Others*, the sword of Damocles comes to signify a call to arms against homophobia. The sword of Damocles is represented by a white banner with the letters ‘§175’ written on it, which quite literally hangs above the procession of men. The sword or banner serves to visualise the imminent threat as each man passes below, but it is also a clarion call against §175. We can also understand this vision of the past as an expression of hope for the future in which homosexuality is reclaimed as an identity proudly written on a banner, in a move constituting what Foucault called ‘reverse discourse’.^42^ The homosexual, subjected to the criminal law, is called upon to use his subjection to turn against the oppressor. As such, the historical vignette seems to explore the power of identity politics and its usefulness for the sexological project.

By using the power of the new medium of film to represent a complex vision of ancestral genealogy in this way, Hirschfeld, in his collaboration with Oswald, uses film to paint a picture of homosexual historical identity as a succession of extraordinary, stable individuals injured by intolerance and exclusion but with the power to come together as a community to fight for a better, more just future. In this way, *Different from the Others* exemplifies the use of the past as affirmative history, as described by Doan.

Yet, there are also ways in which this affirmative history is complicated in the film. In *Feeling Backward*, Heather Love, much like Doan, questions the efficacy and usefulness of ‘effective’ and affirmative history. Instead, Love turns her focus on ‘affective history’, asking about the kinds of relations scholars of queer studies might want to have with the subjects they study.^43^ Love is particularly interested in negative affect, such as need, aversion, and longing, which pervades the relationships established between past and present.^44^ Here, she also reacts against ‘the need to turn the difficulties of gay, lesbian, and transgender history to good political use in the present’ as ‘redemptive narratives’.^45^ Seeing history only as an effective history of progress in this way leaves a blind spot in the ‘archive of feeling’, a term she borrows from Ann Cvetkovich.^46^ Queer history's archive of feeling is filled with emotions such as ‘nostalgia, regret, shame, despair, *ressentiment*, passivity, escapism, self‐hatred, withdrawal, bitterness, defeatism’, and ‘shyness, ambivalence, failure, melancholia, loneliness, regression, victimhood, heartbreak, antimodernism, immaturity’.^47^ In *The Hirschfeld Archives*, which considers the lesser‐known works of Magnus Hirschfeld, Heike Bauer builds on such a focus of negative affect as proposed by Love. Bauer argues that in order to study modern queer culture, we must accept that ‘silences, gaps, and omissions, as much as concrete evidence, tell a story about past lives and the norms and power relations that shaped them’.^48^ By excluding negative affect, which is an undeniable fact of queer history and its subjection to violence, exclusion and denial, effective histories risk producing the very gap in the queer archive which queer history seeks to uncover.

In *Different from the Others*, the most striking failure of the affirmative, celebratory view of queer history becomes visible when considering Körner's action triggered by the historical vision. The vision of homosexual kinship across time, although it may aim to create a vision for the future, does not hold a future for Körner. Körner dies. The historical vision of homosexual ancestry plays a significant part in this death. It is upon seeing, in his mind's eye, the procession of men from all times and places which he, as a victim of prejudice, must finally join, that Körner truly understands his grave situation. It is by witnessing this desperate vision that his fate is sealed and he poisons himself. Here, the historical vision flips over into a nightmarish realisation that haunts Körner and that finally turns him into a haunting presence himself as the final member of the procession. As Körner realises the inevitability of his own death, the *Danse Macabre* topos of the scene is revealed.

When Körner finally joins the procession as its most recent member, a striking failure of his historical vision is uncovered: the queer genealogy across time, which the historical vignette seems to offer, offers no real community. These men are dispersed across time and space and each person remains isolated in their individuality. As Körner joins the parade as the final member, his extraordinary talent as famous musician among other accomplished men is no longer a badge of honour but a curse that singled him out as the final member in this deadly parade. Körner's death reveals everything the historical vision seeks to establish – community, individuality, extraordinariness, a hopeful future – not as a vision, but an illusion. In a final act of fatalistic cruelty, the historical scene, which is meant to secure and affirm Körner's male homosexual identity, is now lost. All that remains today is a production still, which stands in for the historical scene. As such, the film's attempt to reach back to the past to create a noble and stable genealogy is thwarted. This threatens to undermine Hirschfeld's sexological project to use history affirmatively to secure identity categories.

By turning towards film, sexology entrusts its aims of effective history and popularisation to a medium that could turn against its maker. As Andrew J. Webber notes, in the early years of film, cinema provided ‘a potent new medium for the projection of fantasies, but it is also shadowed by anxieties over the maintenance and control of those fantasies’.^49^ While the historical vignette in *Different from the Others* can be seen as one such fantasy, which seeks to establish an affirmative history of homosexuality, we can equally see how these fantasies can spiral out of control and exceed their intended meaning. This risk of excess, failure or unpredictability, however, reveals something fundamental about the sexual rights efforts of Hirschfeld's particular brand of sexology: that failure, loss, and a message not received form part of the sexological project. The film performs the negative affects of gay shame, ambivalence about the past, victimhood, heartbreak, and a wealth of those that Love describes, as foundational to the affective archive of queer history. For the sexual rights efforts of Hirschfeld's sexological project, so much relied on the affective force of effective history and popularisation. The film reveals the fear that cannot be contained, that the message might fall flat, that it would not lead to greater acceptance, but censorship; that it would not achieve the removal of §175 from the German Penal Code, which remained for many decades to come. Acknowledging the affective gaps in the queer archive of feeling presented in *Different from the Others*, we might understand the film not only as a gay rights film that forms part of a long history of sexual rights activism, foreshadowing a better time to come. In sexology's complex challenge to make film work for its own purposes and to use film to visualise its vision of effective history, we can see that queer history itself takes on the shape of the sort of dual personality Hirschfeld describes in *Berlin's Third Sex*, as beacon and death, vision and illusion, an archive of feelings both hopeful and devastated that everything it creates might one day be unravelled.

## Conclusion: Let there be light!

This discussion of the challenges of translating sexological and sexual rights agendas into film is not to say that *Different from the Others* is a failed film project, or that Hirschfeld misunderstood the medium of film. Rather, this article highlights the complexity of the new medium of film, which was chosen by Hirschfeld to popularise his sexological project, but which altered intended aims in unexpected and contradictory ways.

In its investigation of the relationship between sexology and film, this article was limited to a discussion of *Different from the Others*. Future work on the relationship between sexology and film would benefit from an investigation into Hirschfeld's further involvement with film. After the completion of *Different from the Others*, its tumultuous reception and the eventual reintroduction of film censorship, Hirschfeld assisted in the making of two other films entitled *Das Recht auf Liebe* (*The Right to Love*) (Jacob Fleck and Luise Fleck, 1930) and *Vererbte Triebe* (*Inherited Drives*) (Gustav Ucicky, 1929).^50^ It is possible that Hirschfeld was also involved in the making of another film about transvestites entitled *Mann oder Weib* (*Man or Woman*) (director unknown, 1922), which was shown at his Institute of Sexology in 1922.^51^ The film *Aus eines Mannes Mädchenjahren* (*A Man's Maiden Years*) (Julius Rode and Paul Legband, 1919), described as a ‘sexuell‐ethischer Film’ (sexual ethics film), was an adaptation (although not a particularly faithful one) of an autobiography of the same name written by one of Hirschfeld's former patients.^52^ In 1928, Hirschfeld became an honorary president of the magazine *Film und Volk* (*Film and the People*, 1928–30). And when visiting California as the final stop on his US tour in 1931, he travelled to Hollywood to meet up with the director Paul Fejos at MGM Studios.^53^

Future research into the relationship between sexology and film would also benefit from examining the relationship of other sexologists with film and the promises and anxieties projected onto this medium. This includes cinema critics such as Albert Moll, who saw dangers in the suggestive power of film. It also includes those who used film enthusiastically, such as Iwan Bloch, who worked as consultant for the second and third part of *Let There be Light!* and the American sex educator Margaret Sanger, who produced a film entitled *Birth Control* (1917). To further understand the hopes and fears sexologists had when they turned towards film, it would be useful to investigate how sexology's engagement with film compared with its use by other human sciences. A film featuring Ernst Haeckel, which Hirschfeld mentions as inspiration for his own engagement with the medium, as well as *Der Steinach‐Film* (1922), co‐written by the Austrian endocrinologist Eugen Steinach, whose work inspired Hirschfeld, or the film plans of Charlotte Wolff, a psychotherapist interested in the study of sexuality and Hirschfeld's first biographer, who sought to turn her research into a film about palmistry, would be interesting examples here.^54^

My hope is that this article will not only inspire further research on the relation between sexology and film, but also contribute to the burgeoning field of research on ‘useful cinema’, wherein the medium of film is engaged by scientists and social scientists, among others, for what they considered useful purposes, beyond those of art or purely escapist entertainment.^55^

## Notes

1 Magnus Hirschfeld, ‘Aus der Bewegung’, *Jahrbuch für sexuelle Zwischenstufen* 19 (1919), pp. 3–68, here p. 4.

2 Research for this article was funded by the Wellcome Trust under grant numbers 106653/Z/14/Z and 106654/Z/14/Z. In compliance with Open Data requirements, I declare that the research data supporting this publication are provided within this paper.

3 For other German films from the interwar period that imply homosexuality, see Richard Dyer, *Now You See It: Studies on Lesbian and Gay Film* (London: Routledge, 1990), pp. 23–62. For a more general discussion of gender and sexuality in Weimar Germany, see Richard McCormick, *Gender and Sexuality in Weimar Modernity: Film, Literature, and ‘New Objectivity’* (New York: Palgrave, 2001).

4 The reasons for this loss are complex. Whereas some of Oswald's films still exist today, at least in part, his sexual hygiene films were often threatened by censors, who asked for certain scenes to be cut. This accounts at least for the fragmentary nature of *Different from the Others*. Another contributing factor may have been that Oswald's film company declared bankruptcy in 1926, which may have meant that there was no longer adequate storage for these film reels.

5 The version referred to here is the third extended version by Edition Filmmuseum from 2011.

6 Scholarship which understands sexology predominantly as a medical field include, for example, Chiara Beccalossi, *Female Sexual Inversion: Same‐Sex Desires in Italian and British Sexology, c.1870–1920* (Basingstoke: Palgrave Macmillan, 2012); Ivan Crozier, ‘Havelock Ellis, Eonism and the Patient's Discourse; or, Writing a Book About Sex’, *History of Psychiatry* 11 (2000), pp. 112–54; Vernon Bullough, *Science in the Bedroom: A History of Sex Research* (New York: Basic Books, 1994).

7 See, for example, Heike Bauer, *English Literary Sexology: Translations of Inversion, 1860–1930* (Basingstoke: Palgrave Macmillan, 2009); Anna Katharina Schaffner, *Modernism and Perversion: Sexual Deviance in Sexology and Literature, 1850–1930* (Basingstoke: Palgrave Macmillan, 2012); Kate Fisher and Jana Funke, ‘British Sexual Science beyond the Medical: Cross‐Disciplinary, Cross‐Historical, and Cross‐Cultural Translations’, in Heike Bauer (ed.), *Sexology and Translation: Cultural and Scientific Encounters Across the Modern World* (Philadelphia: Temple University Press, 2015), pp. 95–114; Ina Linge, ‘Gender and Agency between “Sexualwissenschaft” and Autobiography: The Case of N. O. Body's *Aus eines Mannes Mädchenjahren*’, *German Life and Letters* 68 (2015), pp. 387–405.

8 The Sexological Institute in Prague, founded in 1921, was the first sexological institute to be affiliated with a university (the Medical School of Charles University). See Kateřina Lišková, ‘“Now you see them, now you don't”: Sexual Deviants and Sexological Expertise in Communist Czechoslovakia’, *History of the Human Sciences* 29 (2016), pp. 49–74; George S. Viereck, ‘Hirschfeld: The Einstein of Sex’, in his *Glimpses of the Great* (London: Duckworth, 1930), pp. 240–59.

9 By arguing that homosexuality is a natural state of being, Hirschfeld tries to disprove the idea that homosexuality is a crime. For debates about homosexuality as crime, see Robert D. Tobin, ‘Introduction: 1869 – Urnings, Homosexuals, and Inverts’, in his *Peripheral Desires: The German Discovery of Sex* (Philadelphia: University of Pennsylvania Press, 2015), pp. 1–25.

10 Magnus Hirschfeld, *Die Transvestiten: Eine Untersuchung über den erotischen Verkleidungstrieb* (Berlin: Alfred Pulvermacher & Co., 1910).

11 Homosexual acts between women were not criminalised under §175.

12 Atina Grossmann, *Reforming Sex: The German Movement for Birth Control and Abortion Reform* (Oxford: Oxford University Press, 1995).

13 For a detailed account of the Harden‐Eulenburg affair, see Norman Domeier, *The Eulenburg Affair: A Cultural History of Politics in the German Empire* (Rochester: Camden House, 2015).

14 Anton Kaes, ‘Film in der Weimarer Moderne’, in Wolfgang Jacobsen, Anton Kaes and Hans Helmut Prinzler (eds), *Geschichte des deutschen Films* (Weimar: Metzler, 1993), pp. 39–100, here p. 39.

15 Peter Broks, *Understanding Popular Science* (Maidenhead: Open University Press, 2006), pp. 50–72.

16 Magnus Hirschfeld, *Berlin's Third Sex*, tr. James J. Conway (Berlin: Rixdorf, 2017), p. 9.

17 Hirschfeld, *Berlin's Third Sex*, pp. 47–8.

18 Magnus Hirschfeld, ‘Das Ergebnis der statistischen Untersuchungen über den Prozentsatz der Homosexuellen’, *Jahrbuch für sexuelle Zwischenstufen mit besonderer Berücksichtigung der Homosexualität* 6 (1904), pp. 109–78, here p. 170.

19 Erwin J. Haeberle, ‘Introduction’, in Magnus Hirschfeld, *Die Homosexualität des Mannes und des Weibes* (Berlin: de Gruyter, 1984), pp. v–xxxi.

20 Jill S. Smith, ‘Richard Oswald and the Social Hygiene Film: Promoting Public Health or Promiscuity?’, in Christian Rogowski (ed.), *The Many Faces of Weimar Cinema: Rediscovering Germany's Filmic Legacy* (Rochester: Camden House, 2010), pp. 13–30, here p. 14.

21 Smith, ‘Richard Oswald’, p. 14.

22 Smith, ‘Richard Oswald’, p. 14.

23 For a brief biography of Bloch, see Günter Grau, *Iwan Bloch: Hautarzt – Medizinhistoriker – Sexualforscher* (Berlin: Hentrich & Hentrich, 2007).

24 J. T. Soister, *Conrad Veidt on Screen: A Comprehensive Illustrated Filmography* (Jefferson: McFarland & Company, 2002), p. 47.

25 Cited in Hirschfeld, ‘Aus der Bewegung’, p. 34.

26 Rainer Herrn, ‘On the History of Biological Theories of Homosexuality’, *Journal of Homosexuality* 28 (1995), pp. 31–56.

27 Smith, ‘Richard Oswald’, p. 25.

28 Smith, Richard Oswald’, p. 14.

29 Peter Jelavich, ‘Metamorphosis of Censorship in Modern Germany’, *German Politics & Society* 27 (1995), pp. 25–35, here p. 26.

30 For a detailed account of the censorship history of *Different from the Others*, see James D. Steakley, *Anders als die Andern: Ein Film und seine Geschichte* (Hamburg: Männerschwarm Verlag, 2007) and James D. Steakley, ‘Cinema and Censorship in the Weimar Republic: The Case of *Anders als die Andern*’, *Film History* 11 (1999), pp. 181–203.

31 Dyer, *Now You See It*, p. 29.

32 Hirschfeld, *Berlin's Third Sex*, p. 18.

33 The intertitles were translated from a Ukrainian fragment of the film. Steakley, *Ein Film und seine Geschichte*, pp. 9–15.

34 Magnus Hirschfeld, ‘Leonardo da Vinci’, *Jahrbuch für sexuelle Zwischenstufen* 10 (1909/1910), pp. 421–6.

35 Numa Prätorius [pseudonym of Eugène Wilhelm], ‘Oskar Wilde’, *Jahrbuch für sexuelle Zwischenstufen* 3 (1901), pp. 265–72.

36 Sappho was a poet from Lesbos in ancient Greece who is often considered a symbol of female homosexuality. Richard Dyer implies that the procession in *Different from the Others* also included women from history. However, I have not been able to find any evidence for this. Dyer, *Now You See It*, p. 27.

37 On John Addington Symonds, Havelock Ellis and the uses of ancient Greece, see Jana Funke, ‘“We cannot be Greek now”: Age Difference, Corruption and the Making of Sexual Inversion’, *English Studies: A Journal of English Language and Literature* 94 (2013), pp. 139–53. On the uses of Rome in British sexology, see Jana Funke and Rebecca Langlands, ‘Uses of Rome in English Sexology’, in Jennifer Ingleheart (ed.), *Romosexuality: The Reception of Rome and the Construction of Western Homosexual Identities* (Oxford: Oxford University Press, 2015), pp. 109–25. On Iwan Bloch and historical sexology see Ralph Leck, *Vita Sexualis: Karl Heinrich Ulrichs and the Origins of Sexual Science* (Urbana: University of Illinois Press, 2016). For sexology in the context of reception studies, see Sebastian Matzner, for example ‘From Uranians to Homosexuals: Philhellenism, Greek Homoeroticism and Gay Emancipation in Germany, 1835–1915’, *Classical Receptions Journal* 2 (2010), pp. 60–91.

38 The word he uses is ‘Uranian’, another term that was used to describe homosexuals. Rainer Herrn, ‘Ein historischer Urning: Ludwig II von Bayern im psychiatrisch‐sexualwissenschaftlichen Diskurs und in der Homosexuellenbewegung des frühen 20. Jahrhunderts’, in Katharina Sykora (ed.), *Ein Bild von einem Mann: Ludwig II von Bayern, Konstruktion und Rezeption eines Mythos* (Frankfurt: Campus, 2004), pp. 48–89, here pp. 51–2. For more on Moll, see Volkmar Sigusch, ‘The Sexologist Albert Moll – between Sigmund Freud and Magnus Hirschfeld’, *Medical History* 56 (2012), pp. 184–200.

39 Tobin, *Peripheral Desires*, p. 126.

40 Laura Doan, *Disturbing Practices: History, Sexuality, and Women's Experience of Modern War* (Chicago: University of Chicago Press, 2013), p. 58.

41 The historical scene is marked by a certain level of historical inaccuracy, or perhaps artistic licence. §175 had been introduced in the 1870s and most of the characters depicted in the procession were alive before that time and would not have been affected by this change in the German law.

42 Michel Foucault, *The History of Sexuality: The Will to Knowledge*, tr. Robert Hurley (1976; repr. London: Penguin, 1998).

43 Heather Love, *Looking Backward: Loss and the Politics of Queer History* (Cambridge: Harvard University Press, 2009), p. 31.

44 Love, *Looking Backward*, pp. 31–2.

45 Love, *Looking Backward*, pp. 104, 119.

46 Ann Cvetkovich, *An Archive of Feelings: Trauma, Sexuality, and Lesbian Cultures* (Durham: Duke University Press, 2003). In Love, *Looking Backward*, p. 4.

47 Love, *Looking Backward*, p. 4, p. 146.

48 Heike Bauer, *The Hirschfeld Archives: Violence, Death, and Modern Queer Culture* (Philadelphia: Temple University Press, 2017), p. 9.

49 Andrew J. Webber, ‘The Manipulation of Fantasy and Trauma in *Orlacs Hände*’, in Katrin Kohl and Ritchie Robertson (eds), *Words, Texts, Images* (Bern: Peter Lang, 2002), pp. 153–74, here p. 153.

50 Magnus Hirschfeld, *Geschlechtskunde auf Grund dreißigjähriger Forschung und Erfahrung bearbeitet*, vol. IV (Stuttgart: Julius Püttmann, 1930), p. 902.

51
*Mann oder Weib* was produced by the film production company Universum Film AG (UFA) , with the support of the Institute of Sexology. The film is considered lost and little is known about its content. See Manfred Herzer, *Magnus Hirschfeld und seine Zeit* (Berlin: de Gruyter, 2017), pp. 333–4.

52 For more information about the autobiographical text from which this film takes its name, see Linge, ‘Gender and Agency’.

53 Paul Fejos’ film *The Last Performance* (1927) had starred Conrad Veidt a few years previously. See Bauer, *The Hirschfeld Archives*, p. 108.

54 Hirschfeld, ‘Aus der Bewegung’, p. 6; Wellcome Collection PSY/WOL/2/27.

55 In ‘useful cinema’ as defined by Charles R. Acland and Haidee Wasson, ‘cameras, films, and projector are taken up and deployed variously – beyond questions of art and entertainment, in order *to do something* in particular’. See Charles R. Acland and Haidee Wasson (eds), *Useful Cinema* (Durham and London: Duke University Press, 2011), p. 3. For a discussion of ‘useful cinema’ in early Germany, see Scott Curtis, *The Shape of Spectatorship: Art, Science, and Early Cinema in Germany* (New York: Columbia University Press, 2015).

